# Tildrakizumab in Managing Psoriasis with Involvement of Difficult-to-Treat Areas: A Multicenter Real-Life Retrospective Study

**DOI:** 10.3390/jcm15020631

**Published:** 2026-01-13

**Authors:** Ruggero Cascio Ingurgio, Angela Alfano, Elena Matteodo, Luciano Ibba, Luigi Gargiulo, Giovanni Paolino, Santo Raffaele Mercuri, Andrea Carugno, Nicola Zerbinati, Stefano Bighetti, Antonio Costanzo, Alessandra Narcisi, Mario Valenti

**Affiliations:** 1 Dermatology Unit, IRCCS Humanitas Research Hospital, 20089 Milan, Italy; ruggero.cascioingurgio@humanitas.it (R.C.I.); angela.alfano@humanitas.it (A.A.); elena.matteodo@humanitas.it (E.M.); luciano.ibba@humanitas.it (L.I.); luigi.gargiulo@humanitas.it (L.G.); antonio.costanzo@hunimed.eu (A.C.); alessandra.narcisi@humanitas.it (A.N.); 2 Department of Biomedical Sciences, Humanitas University, 20089 Milan, Italy; 3 Dermatology and Cosmetology Unit, IRCCS San Raffaele Hospital, 20089 Milan, Italy; paolino.giovanni@hsr.it (G.P.); mercuri.santoraffaele@hsr.it (S.R.M.); 4 Dermatology Clinic, IRCCS San Raffaele Hospital, Vita-Salute San Raffaele University, 20132 Milan, Italy; 5 Dermatology Unit, Department of Medicine and Surgery, University of Insubria, 21100 Varese, Italy; andrea.carugno@uninsubria.it (A.C.); nzerbinati@centro-medico.it (N.Z.); 6 Dermatology Department, University of Brescia, Azienda Socio-Sanitaria Territoriale (ASST) Spedali Civili di Brescia, 25123 Brescia, Italy; s.bighetti@unibs.it

**Keywords:** psoriasis, tildrakizumab, real-life evidence, scalp psoriasis, genital psoriasis, nail psoriasis, palmoplantar psoriasis, IL-23 inhibitors

## Abstract

**Background**: Psoriasis involving difficult-to-treat anatomical areas, such as the scalp, genitalia, fingernails, and palmoplantar regions, carries a disproportionate disease burden and often requires systemic therapy. In this context, real-life data comparing the long-term effectiveness of tildrakizumab 100 mg versus 200 mg in patients with difficult-to-treat psoriasis remain limited. **Methods**: This multicenter retrospective observational study included adult patients in three Italian dermatology centers. Global efficacy endpoints included PASI75, PASI90, PASI100, and absolute PASI ≤ 2 at weeks 16, 32, 52, and 104. Site-specific effectiveness was assessed as complete clearance (PGA = 0) in patients with baseline involvement (PGA ≥ 2) of difficult-to-treat areas. Outcomes were described by dose. **Results**: 183 patients were included (100 mg: n = 89; 200 mg: n = 94). Patients receiving 200 mg had higher baseline BMI and were more frequently biologic-experienced. At week 104, PASI75 was achieved by 94.2% of patients receiving 100 mg and 94.7% receiving 200 mg, while PASI90 and PASI100 were achieved by 82.7% vs. 57.9% and 48.1% vs. 47.4%, respectively. Clearance of difficult-to-treat areas improved progressively across all sites. Scalp and genital psoriasis showed higher and earlier clearance rates, whereas nail and palmoplantar psoriasis showed slower and more heterogeneous responses. No consistent dose-dependent advantage emerged, despite less favorable baseline characteristics in the 200 mg group. **Conclusions**: Over 104 weeks, tildrakizumab showed sustained long-term effectiveness in both global disease control and difficult-to-treat areas. The 200 mg dose, used in a more difficult-to-treat population, achieved comparable long-term outcomes, supporting dose optimization in clinical practice.

## 1. Introduction

Psoriasis is a chronic, immune-mediated inflammatory skin disease characterized by a relapsing–remitting course. It affects approximately 2–3% of the world population. The disease is associated with a substantial physical and psychological burden, with significant impairment in health-related quality of life, work productivity, and social functioning [1,2]. While disease severity has traditionally been assessed using global measures such as the Psoriasis Area and Severity Index (PASI), it is increasingly recognized that lesion localization plays a crucial role in determining disease impact and therapeutic needs.

In particular, involvement of so-called difficult-to-treat areas like scalp, genital region, fingernails, and palmoplantar surfaces, represents a major challenge in the management of psoriasis [3,4]. Psoriasis affecting these sites is often associated with pain, pruritus, bleeding, functional impairment, and marked psychosocial distress. Even when overall body surface area involvement is limited, lesions in these areas may affect patients’ quality of life and lead to treatment dissatisfaction [5,6,7]. As a result, patients with predominant involvement of difficult anatomical sites may require systemic therapy despite relatively low PASI scores.

Topical treatments are the first-line option for localized psoriasis; however, their effectiveness in difficult-to-treat areas is frequently limited by poor penetration, local irritation, and suboptimal adherence [8]. Conventional systemic therapies can also be inadequate or poorly tolerated in these patients. Consequently, biologic agents targeting key inflammatory pathways play a central role in the management of moderate-to-severe psoriasis, including cases with predominant involvement of sensitive anatomical regions.

Beyond their effects on skin clearance, biologic therapies have also demonstrated meaningful improvements in patient-reported outcomes, including quality of life, daily functioning, and treatment satisfaction. This aspect is particularly relevant in patients with difficult-to-treat psoriasis, in whom symptom burden is often underestimated by conventional severity scores. Scalp [9] and genital involvement [10,11,12], for instance, have been consistently associated with higher levels of pruritus, embarrassment, and sexual dysfunction, whereas nail [13,14] and palmoplantar psoriasis [15,16] may cause pain and functional limitations that significantly interfere with daily activities and work performance.

Among biologic therapies, inhibitors of the interleukin (IL)-23/IL-17 axis have demonstrated high efficacy and favorable safety profiles. IL-23 plays a pivotal role in the differentiation and maintenance of Th17 cells, which are key drivers of chronic inflammation in psoriasis. Selective inhibition of IL-23 has been associated with durable clinical responses and sustained disease control, supporting its use as a long-term treatment strategy [17,18]. Importantly, the clinical behavior of psoriasis in difficult anatomical sites may differ from that observed on the trunk and limbs, often requiring prolonged exposure to biologic therapy to achieve optimal disease control.

Tildrakizumab is a humanized monoclonal antibody selectively targeting the p19 subunit of IL-23. In pivotal phase III trials [19], tildrakizumab demonstrated significant efficacy compared with placebo and etanercept, with sustained responses observed over extended follow-up periods [20]. Based on these data, tildrakizumab was approved at a standard dose of 100 mg, with the option of dose escalation to 200 mg in selected patients. However, randomized controlled trials were not specifically designed to assess outcomes in difficult-to-treat areas, and direct comparisons between the two dosing regimens remain limited.

In real-life clinical practice, dose escalation of biologic therapies is frequently considered in patients perceived as more difficult to treat, such as those with higher body mass index (BMI), prior biologic exposure, or predominant involvement of challenging anatomical sites [21,22]. Real-life studies have shown that these factors may influence treatment response and persistence, underscoring the importance of observational data to complement evidence derived from clinical trials. Recent real-life analyses of other biologic agents have further highlighted the value of long-term observational studies in understanding treatment effectiveness in specific patient subgroups and anatomical regions [23,24].

Despite the growing use of tildrakizumab in routine practice, real-life data directly comparing the long-term effectiveness of the 100 mg and 200 mg doses are scarce, particularly with regard to difficult-to-treat areas and extended follow-up beyond one year. Moreover, the impact of prior biologic exposure on treatment outcomes with different tildrakizumab dosing regimens remains poorly characterized.

The present multicenter retrospective study was therefore designed to evaluate the long-term real-life effectiveness of tildrakizumab 100 mg versus 200 mg in patients with moderate-to-severe psoriasis involving difficult-to-treat areas. By analyzing data from three Italian dermatology centers over a follow-up period of up to 104 weeks, we aimed to assess global disease severity outcomes, site-specific responses in scalp, genital, nail, and palmoplantar psoriasis, and exploratory differences according to prior biologic exposure.

## 2. Materials and Methods

This was a multicenter, retrospective, observational study conducted across three Italian tertiary referral dermatology centers with expertise in the management of moderate-to-severe psoriasis. The study was designed to reflect routine clinical practice and to provide real-life evidence on the long-term effectiveness of tildrakizumab in patients with difficult-to-treat psoriasis.

Clinical data were extracted from institutional databases and medical records. Given the retrospective and non-interventional nature of the study, no predefined treatment protocols or visit schedules were imposed. All clinical assessments were performed as part of standard patient care, reflecting real-life therapeutic decision-making.

Adult patients (≥18 years) with a confirmed diagnosis of plaque psoriasis who initiated treatment with tildrakizumab between January 2020 and June 2025 were eligible for inclusion. Follow-up duration varied according to treatment initiation date, with a maximum available follow-up of 104 weeks. Patients were included if they had documented involvement of at least one difficult-to-treat anatomical site—defined as scalp, genitalia, fingernails, or palmoplantar areas—and at least one post-baseline follow-up assessment.

Both biologic-naïve and biologic-experienced patients were eligible. Prior biologic exposure was heterogeneous and reflected routine clinical practice. No stratification according to the number or class of previously administered biologic agents was performed due to sample size limitations and the retrospective nature of data collection. Prior biologic exposure was defined as previous treatment with at least one biologic agent approved for psoriasis. Patients with incomplete baseline data or without any follow-up visits were excluded.

Tildrakizumab was administered according to standard clinical practice at a dose of either 100 mg or 200 mg, with subcutaneous injections at weeks 0 and 4 and every 12 weeks thereafter. Dose selection was at the discretion of the treating physician, based on disease severity, patient characteristics, previous treatment history, and clinical judgment.

Dose escalation from 100 mg to 200 mg was permitted during follow-up in cases of inadequate clinical response. Overall, 17 patients escalated from 100 mg to 200 mg during the observation period. Among these patients, dose escalation was driven by secondary loss of efficacy in 13 cases and primary lack of response in 4 cases. These patients were subsequently analyzed within the 200 mg group for all outcome assessments.

Baseline data included age, sex, body mass index (BMI), disease duration, baseline PASI score, presence of psoriatic arthritis, cardiometabolic comorbidities, prior biologic exposure, and involvement of difficult-to-treat areas.

Overall disease severity was assessed using the Psoriasis Area and Severity Index (PASI). Efficacy endpoints included PASI75, PASI90, PASI100 and absolute PASI ≤ 2 at weeks 16, 32, 52, and 104, when available [25,26].

Site-specific disease severity was evaluated using Physician’s Global Assessment (PGA) scores for scalp, genitalia, fingernails, and palmoplantar areas [27,28]. For site-specific analyses, only patients with baseline involvement (PGA ≥ 2) for the respective site were included. Site-specific effectiveness was assessed as complete clearance (PGA = 0) at weeks 16, 32, 52, and 104.

Given the significant imbalance in prior biologic exposure between treatment groups at baseline, an exploratory subgroup analysis according to biologic-naïve versus biologic-experienced status was performed. Due to heterogeneity and limited sample size, BMI-stratified efficacy analyses were not included in the main analysis.

Continuous variables were summarized as mean ± standard deviation (SD), while categorical variables were expressed as absolute numbers and percentages. Comparisons between treatment groups (tildrakizumab 100 mg vs. 200 mg) for continuous variables were performed using Student’s *t*-test, whereas categorical variables were compared using Pearson’s chi-square test or Fisher’s exact test, as appropriate.

Efficacy endpoints were evaluated descriptively at each follow-up time point (weeks 16, 32, 52, and 104). Global efficacy outcomes included the proportion of patients achieving PASI75, PASI90, PASI100 and absolute PASI ≤ 2. Site-specific effectiveness was assessed as the proportion of patients achieving complete clearance (PGA = 0) among those with baseline site involvement (PGA ≥ 2) for each difficult-to-treat area.

Binary efficacy outcomes were reported descriptively as the number and percentage of patients achieving each endpoint at the respective time points and were not analyzed using inferential statistical testing, given the observational nature of the study and the heterogeneity of follow-up availability. *p*-Values were therefore applied only to baseline comparisons between treatment groups.

Analyses at each time point were conducted on available cases, using an as-treated approach. No intention-to-treat or sensitivity analyses were performed, as treatment discontinuations and missing follow-up data reflect routine clinical practice in this retrospective real-life setting. All statistical analyses were performed using STATA/SE 17.0 software (StataCorp, College Station, TX, USA). Due to the retrospective nature of data collection, not all parameters were consistently available at each follow-up visit. Missing data were therefore not imputed. In addition, not all patients had completed the 104-week follow-up at the time of data cutoff, as treatment initiation occurred at different time points in routine clinical practice. Treatment discontinuations were recorded when available and were not systematically analyzed for efficacy outcomes. All patients provided written informed consent for the collection and use of clinical data within routine care, in accordance with local regulations on patient privacy. The study was conducted in accordance with the principles of the Declaration of Helsinki and its subsequent amendments.

## 3. Results

A total of 183 patients took part in the analysis, 89 patients received tildrakizumab 100 mg and 94 patients 200 mg. Baseline demographic and clinical characteristics are summarized in Table 1.

The two treatment groups were comparable in terms of age, disease duration, baseline PASI score, and prevalence of psoriatic arthritis and cardiovascular comorbidities. Patients treated with tildrakizumab 200 mg tended to have a higher BMI than the 100 mg group (27.5 ± 5.7 vs. 24.7 ± 3.9 kg/m^2^, *p* = 0.0001) and were more frequently biologic-experienced (26.6% vs. 10.1%, *p* = 0.007). These findings suggest that dose intensification was preferentially adopted in patients perceived as more difficult to treat in routine clinical practice. Baseline involvement in challenging regions was prevalent across both groups, with the scalp emerging as the most commonly affected area, followed by the genital region, fingernails, and palmoplantar region (Table 1).

**Figure 1:**
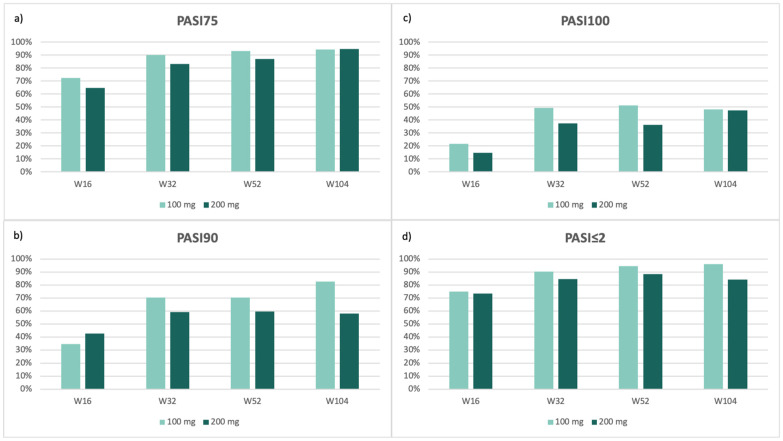
Percentage of patients achieving PASI75 (**a**), PASI90 (**b**), PASI100 (**c**), and absolute PASI ≤ 2 (**d**) at weeks 16, 32, 52, and 104, stratified by tildrakizumab dose (100 mg vs. 200 mg).

Global efficacy outcomes are shown in Figure 1. The number of evaluable patients at each time point for global PASI outcomes and site-specific analyses is reported in Supplementary Table S1. Both treatment groups showed a steady increase in response rates over time for all the endpoints we evaluated. At week 16, PASI75 was achieved by 72.4% of patients receiving 100 mg and 64.9% of those receiving 200 mg (Figure 1a). PASI90 responses were observed in 34.5% and 42.6% (Figure 1b), respectively, while PASI100 was achieved by 21.8% and 14.9% of patients (Figure 1c).

Response rates continued to improve over time in both groups. At week 104, PASI75 exceeded 94% in both cohorts (94.2% for 100 mg and 94.7% for 200 mg) (Figure 1a), while PASI90 was achieved by 82.7% and 57.9% (Figure 1b), and PASI100 by 48.1% and 47.4% (Figure 1c), respectively.

Most patients achieved an absolute PASI ≤ 2 throughout the follow-up period, highlighting the long-term effectiveness of tildrakizumab in routine clinical practice (Figure 1d).

**Figure 2:**
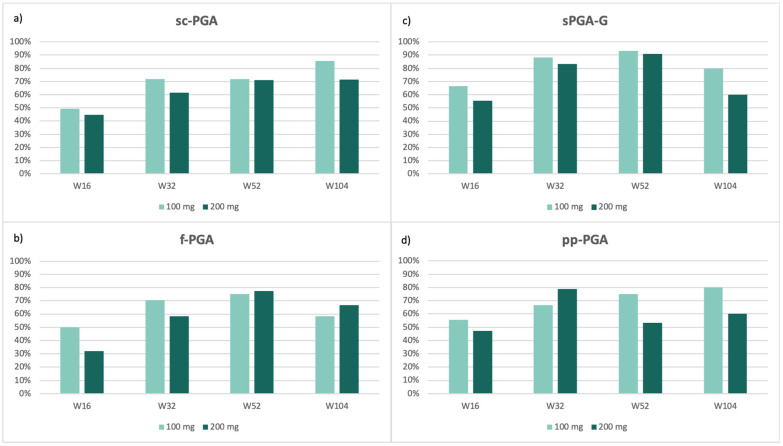
Percentage of patients achieving complete clearance (PGA = 0) among those with baseline site-specific involvement (PGA ≥ 2) in scalp (**a**), fingernails (**b**), genitalia (**c**), and palmoplantar regions (**d**), stratified by tildrakizumab dose (100 mg vs. 200 mg) at weeks 16, 32, 52, and 104.

Site-specific complete clearance (PGA = 0) among patients with baseline site involvement (PGA ≥ 2) is presented in Figure 2. Overall, improvement in difficult-to-treat areas followed a gradual and sustained trajectory, consistent with the known clinical behavior of psoriasis in these areas.

Scalp (Figure 2a) and genital (Figure 2c) psoriasis showed relatively rapid improvement, with high clearance rates already observed at early time points and maintained throughout follow-up. In contrast, fingernail (Figure 2b) and palmoplantar (Figure 2d) psoriasis displayed a slower and more variable response pattern, highlighting the therapeutic challenges associated with these areas. Nevertheless, clinically significant improvements were achieved over time, and sustained PGA 0 responses were achieved in a substantial proportion of patients at long-term follow-up.

Among patients with baseline site involvement (PGA ≥ 2), complete clearance (PGA = 0) increased over time across all anatomical regions (Figure 2a–d). Scalp: 49.2% vs. 44.7% at week 16 (100 vs. 200 mg), increasing to 85.7% vs. 71.4% at week 104 (Figure 2a). Fingernails: 70.6% vs. 58.3% at week 32 and 58.3% vs. 66.7% at week 104 (Figure 2b). Genitalia: high early clearance (88.2% vs. 83.3% at week 32; 93.3% vs. 90.9% at week 52), with lower rates at week 104 (80.0% vs. 60.0%) (Figure 2c). Palmoplantar: 55.6% vs. 47.4% at week 16; an early advantage for 200 mg at week 32 (78.9%), followed by higher clearance in the 100 mg group at week 104 (80.0% vs. 60.0%) (Figure 2d).

Overall, response rates demonstrated a positive trend over time, showing sustained efficacy through week 104 among available cases across both dosing regimens. At later timepoints, particularly at week 104, greater variability was observed, likely reflecting reduced sample size and real-life treatment discontinuations.

Notably, patterns of improvement varied significantly across anatomical regions and timepoints. No consistent correlation regarding dosage amounts was observed, suggesting that site-specific response dynamics play a greater role in therapeutic outcomes rather than a dose-dependent uniform response.

In exploratory analyses stratified by prior biologic exposure, response trajectories appeared broadly comparable between biologic-naïve and biologic-experienced patients, despite the more complex baseline profile of the 200 mg group (higher BMI and greater prior biologic exposure).

## 4. Discussion

This multicenter study provides long-term real-life evidence on the effectiveness of tildrakizumab for psoriasis affecting difficult-to-treat anatomical sites [3,4,5,6,7]. Over a follow-up period of up to 104 weeks, both the 100 mg and 200 mg dosing regimens were associated with sustained improvements in global disease severity and site-specific outcomes [19,20].

One of the main strengths of this study lies in its focus on difficult-to-treat areas, which are often underrepresented in randomized clinical trials despite their significant impact on patient quality of life. Our findings demonstrate that tildrakizumab can achieve durable disease control in scalp, genital, nail, and palmoplantar psoriasis, addressing an important unmet need in routine clinical practice [29].

The two dosing approaches showed similar long-term effectiveness. Importantly, patients receiving the 200 mg dose had higher BMI values and were more frequently biologic-experienced at baseline, factors linked in earlier studies to slower or weaker responses [21,22]. In this context, the comparable outcomes observed between dosing groups are likely to reflect the effectiveness of the higher dose in a more difficult-to-treat population rather than the absence of a dose–response relationship. However, differences in previous biologic exposure between treatment groups reflect real-life prescribing patterns and should be considered when interpreting comparative effectiveness outcomes, particularly in the context of a more difficult-to-treat population receiving the 200 mg dose.

Dose increases from 100 mg to 200 mg were needed in some individuals, predominantly due to secondary loss of efficacy. This finding reflects routine clinical practice and highlights the role of the 200 mg dose as a rescue strategy rather than an upfront intensification. Notably, long-term disease control remained achievable following dose escalation, suggesting that treatment optimization may help sustain clinical benefit over time. While this as-treated analytical approach may theoretically introduce bias favoring the 200 mg group, it reflects real-world dose escalation strategies and clinical decision-making in patients with insufficient response.

The subgroup analysis based on earlier use of biologics adds weight to the idea that past treatments shape how quickly responses occur. Biologic-experienced patients exhibited lower response rates at early time points, particularly for stringent endpoints such as PASI90 and PASI100. However, the attenuation of these differences over longer follow-up suggests that prolonged IL-23 inhibition may overcome initial treatment resistance in a proportion of patients.

The long-term nature of the present study enables a deeper look at how well treatments work over time, not just at first. In routine clinical practice, sustained disease control is often a more relevant outcome than short-term efficacy, particularly in chronic conditions such as psoriasis [30]. The maintenance of high response rates through 104 weeks observed in this cohort supports the role of tildrakizumab as a long-term therapeutic option, even in patients with complex disease profiles.

Comparison with real-life studies of other biologic agents targeting the IL-23/IL-17 axis further contextualizes these findings [31,32]. Previous observational studies have shown that both IL-17 and IL-23 inhibitors can achieve meaningful and sustained responses in difficult-to-treat areas, although response patterns may vary according to anatomical site, patient characteristics, and prior treatment exposure. In this context, the extended follow-up and multicenter real-life design of the present study provide additional evidence on the long-term effectiveness of IL-23 inhibition with tildrakizumab in challenging anatomical localizations.

Taken together, these findings support an individualized treatment approach in psoriasis management. Tailoring biologic dosing based on patient characteristics, disease burden, and response dynamics may optimize long-term outcomes. In particular, patients with higher BMI, prior biologic failure, and extensive involvement of difficult-to-treat areas may represent candidates in whom initiation with the 200 mg dose could be considered, while acknowledging that treatment decisions should remain individualized.

Several limitations should be acknowledged. The retrospective design introduces potential selection bias, and the absence of randomization limits causal inference. Sample sizes for some subgroups and anatomical sites were limited, particularly at later time points, and subgroup analyses were exploratory in nature. In addition, although cardiometabolic comorbidities were recorded at baseline, specific conditions such as diabetes mellitus were not analyzed in detail as effect modifiers, despite their potential influence on disease severity, treatment response, and infection risk. Adverse events, including infections, were not systematically analyzed across dosing regimens due to the retrospective design and incomplete safety reporting in routine clinical databases. This represents an inherent limitation of real-world observational studies and may limit the assessment of dose-related safety differences. Nevertheless, the multicenter design, extended follow-up, and focus on difficult-to-treat areas enhance the clinical relevance of the findings. In addition, analyses were conducted on available cases without imputation of missing data, which may have influenced outcome estimates at later time points. Taken together, these considerations highlight the need for cautious interpretation of comparative findings and reinforce the value of prospective studies integrating metabolic profiling and standardized safety assessments in difficult-to-treat psoriasis.

## 5. Conclusions

In this multicenter real-life cohort, tildrakizumab delivered lasting benefits for individuals with moderate-to-severe psoriasis—particularly affecting areas including the scalp, genitals, nails, and palmoplantar involvement. While both the 100 mg and 200 mg dosages were tested, each maintained steady progress over 104 weeks of follow-up.

The 200 mg regimen was frequently adopted in patients with inadequate response or more complex disease profiles, including those with prior biologic exposure or higher baseline disease burden. Taken together, these findings suggest that flexible tildrakizumab dosing can be effectively applied in real-life clinical practice, supporting its use in the long-term management of psoriasis involving difficult-to-treat areas.

## Figures and Tables

**Table 1:** Baseline demographic and clinical characteristics of the study population according to tildrakizumab dose.

Characteristic	100 mg (n = 89)	200 mg (n = 94)	*p*-Value
Mean ± SD			
Age, years	51.76 ± 16.70	50.04 ± 13.06	0.4367
BMI, kg/m^2^	24.69 ± 3.88	27.54 ± 5.66	**0.0001**
Disease duration, years	16.27 ± 13.21	17.83 ± 13.55	0.4316
PASI baseline	10.36 ± 5.25	11.31 ± 6.50	0.8609
N (%)			
Number of patients	89 (100%)	94 (100%)	
Males	51 (57.3%)	63 (67%)	0.175
PsA	1 (1.1%)	3 (3.2%)	0.339
Scalp involvement	67 (75.3%)	76 (80.8%)	0.362
Genitalia involvement	18 (20.2%)	27 (28.7%)	0.182
Fingernails involvement	18 (20.2%)	25 (26.6%)	0.31
Palmoplantar involvement	9 (10.1%)	19 (20.2%)	0.058
sc-PGA ≥ 3	42 (47.2%)	47 (50%)	0.704
sPGA-G ≥ 3	12 (13.5%)	16 (17%)	0.506
f-PGA ≥ 3	13 (14.6%)	12 (12.8%)	0.717
pp-PGA ≥ 3	4 (4.5%)	11 (11.7%)	0.076
CV comorbidity	37 (41.6%)	38 (40.4%)	0.875
Bio-experienced	9 (10.1%)	25 (26.6%)	**0.007**

Abbreviations: BMI, Body Mass Index; PsA, psoriatic arthritis; PASI, Psoriasis Area and Severity Index; sc-PGA, scalp Physician’s Global Assessment; sPGA-G, genital Physician’s Global Assessment; f-PGA, fingernail Physician’s Global Assessment; pp-PGA, palmoplantar Physician’s Global Assessment; CV, cardiovascular. Values with *p* < 0.005 are highlighted in bold.

## Data Availability

Data are available on request from the authors.

## Supplementary Materials

The following supporting information can be downloaded at: https://www.mdpi.com/article/10.3390/jcm15020631/s1, Table S1: Number of evaluable patients at each time point for global PASI and site-specific outcomes, stratified by treatment dose. For global PASI outcomes, n refers to patients with available PASI data at each time point. For site-specific outcomes, n refers to patients with baseline involvement (PGA ≥ 2) and available PGA assessment at the corresponding visit. No imputation of missing data was performed.

## Abbreviations

The following abbreviations are used in this manuscript:

BMI	Body Mass Index
PsA	Psoriatic Arthritis
PASI	Psoriasis Area and Severity Index
sc-PGA	scalp-specific Physician’s Global Assessment
sPGA-G	static Physician’s Global Assessment of Genitalia
f-PGA	fingernail PGA
pp-PGA	palmoplantar PGA
CV	Cardiovascular
SD	Standard Deviation
IL	Interleukin
