# Supplementary material for: Tildrakizumab in Managing Psoriasis with Involvement of Difficult-to-Treat Areas: A Multicenter Real-Life Retrospective Study
Source: J Clin Med. 2026 Jan 13;15(2):631. doi: 10.3390/jcm15020631 (PMC12841847; doi:10.3390/jcm15020631)
Supplement: Supplementary file 1 [file jcm-15-00631-s001.zip › jcm-4085748-supplementary.pdf]

Table S1. Number of evaluable patients at each time point for global PASI and site-specific outcomes, stratified by treatment dose.

Section	Endpoint/Site	Dose	W16 (n)	W32 (n)	W52 (n)	W104 (n)
**Global PASI**	PASI (all endpoints*)	100 mg	87	81	74	52
**Global PASI**	PASI (all endpoints*)	200 mg	94	83	69	19
**Scalp**	sc-PGA≥2	100 mg	65	60	57	42
**Scalp**	sc-PGA≥2	200 mg	76	65	55	14
**Fingernails**	f-PGA≥2	100 mg	18	17	16	12
**Fingernails**	f-PGA≥2	200 mg	25	24	22	14
**Genitalia**	sPGA-G≥2	100 mg	18	17	15	10
**Genitalia**	sPGA-G≥2	200 mg	27	24	22	5
**Palmoplantar**	pp-PGA≥2	100 mg	9	9	8	5
**Palmoplantar**	pp-PGA≥2	200 mg	19	19	15	5

* Global PASI endpoints include PASI75, PASI90, PASI100 and PASI≤2

For global PASI outcomes, n refers to patients with available PASI data at each time point. For site-specific outcomes, n refers to patients with baseline involvement (PGA≥2) and available PGA assessment at the corresponding visit. No imputation of missing data was performed.
